# The Role of DNA Methylation and Histone Modification in Periodontal Disease: A Systematic Review

**DOI:** 10.3390/ijms21176217

**Published:** 2020-08-27

**Authors:** Ismael Khouly, Rosalie Salus Braun, Michelle Ordway, Bradley Eric Aouizerat, Iya Ghassib, Lena Larsson, Farah Asa’ad

**Affiliations:** 1 Department of Oral and Maxillofacial Surgery, College of Dentistry, New York University, New York, NY 10010, USA; bea4@nyu.edu; 2 Department of Cariology and Comprehensive Care, College of Dentistry, New York University, New York, NY 10010, USA; rb2730@nyu.edu; 3 Department of Periodontics, School of Dental Medicine, University of Pennsylvania, Philadelphia, PA 19104, USA; michelle.a.ordway@gmail.com; 4 Bluestone Center for Clinical Research, New York University College of Dentistry, New York, NY 10010, USA; 5 Department of Periodontics and Oral Medicine, University of Michigan, Ann Arbor, MI 48104, USA; ighassib@umich.edu; 6 Department of Periodontology, Institute of Odontology, The Sahlgrenska Academy at University of Gothenburg, SE-405 30 Göteborg, Sweden; lena.larsson@odontologi.gu.se; 7 Department of Biomaterials, Institute of Clinical Sciences, The Sahlgrenska Academy at University of Gothenburg, SE-405 30 Göteborg, Sweden; farah.asaad@gu.se

**Keywords:** DNA methylation, epigenetics, gene expression, gingival diseases, gingivitis, histone modification, periodontal diseases, periodontitis, systematic review

## Abstract

Despite a number of reports in the literature on the role of epigenetic mechanisms in periodontal disease, a thorough assessment of the published studies is warranted to better comprehend the evidence on the relationship between epigenetic changes and periodontal disease and its treatment. Therefore, the aim of this systematic review is to identify and synthesize the evidence for an association between DNA methylation/histone modification and periodontal disease and its treatment in human adults. A systematic search was independently conducted to identify articles meeting the inclusion criteria. DNA methylation and histone modifications associated with periodontal diseases, gene expression, epigenetic changes after periodontal therapy, and the association between epigenetics and clinical parameters were evaluated. Sixteen studies were identified. All included studies examined DNA modifications in relation to periodontitis, and none of the studies examined histone modifications. Substantial variation regarding the reporting of sample sizes and patient characteristics, statistical analyses, and methodology, was found. There was some evidence, albeit inconsistent, for an association between DNA methylation and periodontal disease. *IL6*, *IL6R*, *IFNG*, *PTGS2*, *SOCS1*, and *TNF* were identified as candidate genes that have been assessed for DNA methylation in periodontitis. While several included studies found associations between methylation levels and periodontal disease risk, there is insufficient evidence to support or refute an association between DNA methylation and periodontal disease/therapy in human adults. Further research must be conducted to identify reproducible epigenetic markers and determine the extent to which DNA methylation can be applied as a clinical biomarker.

## 1. Introduction

Periodontitis is a destructive disease of tooth-supporting tissues, induced by bacterial biofilm [1], which consists mainly of gram-negative, anaerobic, and micro-aerophilic bacteria that can colonize the sub-gingival areas [2,3]. This bacterial biofilm triggers an inflammatory host response influenced by environmental, genetic, and epigenetic factors [4,5,6]. Of interest to the present systematic review, epigenetic modifications can further regulate gene expression without altering the DNA sequence of genes that influence an individual’s immune response [7].

Epigenetic modifications are chemical alterations to DNA and its associated histone proteins, which alter gene expression, but are not encoded in the DNA sequence. Such epigenetic modifications result in chromatin remodeling and subsequent activation (turn on) or inactivation (turn off) of a gene’s expression [8,9].

The most widely studied epigenetic mechanism is DNA methylation, the most commonly evaluated form of which is the covalent addition of methyl groups to the fifth carbon on the cytosine base (5 mC) within the CpG islands of the promoter region of a gene, catalyzed by DNA methyltransferases (DNMTs) [10,11]. Histones, which form nucleosomes that are the building blocks of chromatin, can either be acetylated or methylated. Histone acetylation is controlled by histone acetyltransferases (HATs), which add acetyl groups to histones, and histone deacetylases (HDACs), which remove the acetyl groups [12]. Importantly, both DNA methylation and histone modifications are reversible [13] and linked [10,12].

The majority of studies examining the link between epigenetics and periodontal disease have evaluated the changes in the DNA methylation of genes [14,15] and its regulatory role in the production of cytokines, as these mediators play a key role in periodontal tissue destruction [8,13,16]. Lower levels of DNA methylation of genes expressing pro-inflammatory cytokines have been reported in chronic and aggressive periodontitis patients compared to healthy individuals, resulting in their overexpression in inflamed tissues [17,18,19,20]. Interestingly, hypermethylation of certain genes has also been described in chronic periodontal disease, reflecting a down-regulatory mechanism to prevent unrestricted tissue destruction over time [21].

The effects of conventional periodontal therapy on DNA methylation patterns have been studied as well, reporting a prospective positive effect of non-surgical periodontal therapy on the DNA methylation profile of specific genes [15,22].

Similarly, a relationship between histone modification and periodontal disease was recently reported that suggests a role in the activation of major inflammatory pathways, such as NFkB signaling cascade [23]. Furthermore, a recent study described an increase in messenger RNA (mRNA) expression of several HDACs in periodontitis tissues compared to non-periodontitis tissues [24].

Although the available evidence in the literature on the role of epigenetic mechanisms in periodontal disease, a thorough assessment of the published studies is still needed, to better comprehend the evidence on the relationship between epigenetic changes and periodontal disease, and its treatment. Therefore, we have performed a systematic review to comprehensively assess the association between DNA methylation/histone modification and periodontal disease/therapy in healthy adults.

## 2. Methods and Materials

### 2.1. Study Registration

The review protocol was registered in the prospective international registrar of systematic reviews (PROSPERO) (CRD42018104705).

### 2.2. Reporting Format

A detailed protocol was designed according to guidelines of the Preferred Reporting Items for Systematic Review and Meta-analysis (PRISMA) statement. The clinical questions were organized according to these guidelines [25,26].

### 2.3. PECO Question: Population, Exposure, Comparison, and Outcomes

The focus question for the present systematic review was developed using the participants, exposure, comparisons and outcomes (PECO) criteria.

Are periodontal diseases (outcome) associated with DNA methylation and histone modifications (exposure) compared to non-periodontal diseases/healthy control (comparison) in human adults of any race (participants, type of people)?

### 2.4. Eligibility Criteria

#### 2.4.1. Inclusion Criteria

For inclusion in the review, the studies were required to meet the following criteria:

(1) Human clinical studies, including both interventional and observational studies: Randomized controlled trials, cohort studies, case-control studies, and cross-sectional studies.
(2) Studies that describe either an association between epigenetic marks (global, site-specific or genome wide methylation of DNA) and/or histone modifications (methylation, phosphorylation, acetylation, ubiquitylation, and sumoylation) in healthy control and periodontal disease groups.
(3) Studies that assess epigenetic changes in gingival tissues (epithelial and/or connective tissues).
(4) Cases of periodontitis compared to control can either be of chronic periodontitis (CP) or aggressive periodontitis (AgP).
(5) Studies that compare periodontal/gingival health and periodontitis/gingivitis.

#### 2.4.2. Exclusion Criteria

The exclusion criteria were as follows:

(1) Systematic reviews, case reports, animal trials, letter to editors.
(2) Studies describing epigenetic markers other than DNA methylation and histone modifications, such as noncoding RNAs.
(3) Studies that assess epigenetic changes in in vivo animal studies.
(4) Studies that compare periodontal/gingival health and periodontitis/gingivitis.
(5) Studies that include patients who have systemic diseases, lactating or pregnant patients, and patients with long-term use of anti-inflammatory drugs (i.e., for at least one month prior to conducting the experiment).
(6) Studies that examine outcomes in smokers compared to non-smokers as independent study groups.

### 2.5. Types of Outcomes Measured

#### 2.5.1. Primary Outcome

The primary outcomes assessed were DNA methylation and histone modification

#### 2.5.2. Secondary Outcomes

The secondary outcomes assessed included gene expression, DNA methylation of genes after periodontal therapy, and the association between epigenetics and clinical parameters.

### 2.6. Search Strategy

#### 2.6.1. Electronic Database Search

Literature searches were performed in seven databases, including PubMed, Embase, MEDLINE, CINAHL, Web of Science, Scopus, and SciELO, and included articles published through 15 January 2020. The search was not limited by any restrictions on language or date of publication. Primary and secondary concepts were developed and searched for all databases. Primary and secondary subject headings were selected for each database, specifically, and concepts and subject headings were combined for each of the database searches. The search terms, developed by a medical and dental librarian, are listed in Supplement Materials (Table S1).

#### 2.6.2. Hand Searching

References of relevant papers, published through 15 January 2020, were evaluated to be certain that no appropriate articles were overlooked. In addition, manual searches were conducted for reference lists of selected articles and related review articles. Hand searches were performed for key articles published in key periodontal journals from January 2000 through 15 January 2020, including: Journal of Clinical Periodontology, Journal of Dental Research, Journal of Periodontal Research, Journal of Periodontology, and Oral Health and Preventive Dentistry.

#### 2.6.3. Ongoing and Unpublished Clinical Trials

The US National Institutes of Health Clinical Trials Database: (http://clinicaltrials.gov) and other online databases (www.centerwatch.com/clinicaltrials; www.clinicalconnection.com) were searched for ongoing clinical trials. Finally, unpublished studies in the OpenGrey open-access database were searched.

### 2.7. Data Collection and Analysis

#### 2.7.1. Study Selection

Eligibility assessment was performed through title and abstract analysis and full-text analysis, according to the pre-determined eligibility criteria. Titles and abstracts were screened by two independent reviewers (IK, RB) for possible inclusion in the review; after the initial selection, full-text papers were read in detail by the two independent reviewers (IK, RB) to ensure all inclusion criteria were met. Disagreements were solved by discussion. Reasons for study exclusion were recorded.

#### 2.7.2. Data Extraction and Analysis

Data were extracted by two independent reviewers (RB, MM), using tables that were specifically designed for the present review and modified upon second review process, as required for the presentation of general characteristics and outcomes (Supplement Materials, Table S2). The data extraction tables collected information on general characteristics of the included studies and the methodology and results of the included studies. The extracted data tables were compared and consolidated by the two independent reviewers (RB, MM); the final data extraction tables were reviewed by two reviewers (IK, IG) to ensure accurate data extraction and interpretation of the included studies.

#### 2.7.3. Study Quality

Study quality was assessed independently by two reviewers (IG, RB) using the National Heart, Lung, and Blood Institute’s Quality Assessment Tool for Observation Cohort and Cross-Sectional Studies [27]. The tool contains 14 criteria to determine the study design quality, such as inclusion/exclusion criteria, exposure and outcome terms, etc. The criteria were each rated as either *yes*, *no*, or *not reported.* Then, each of the included studies received overall scores of *good*, *fair*, or *poor.* If more than eight criteria were rated as *yes*, the overall quality rating for the study was determined to be *good*; if 7 or 8 were rated as *yes*, the overall quality rating was determined to be *fair*; and if less than 7 were rated as *yes*, the overall quality was determined to be *poor.* Any disagreements were discussed, and a third reviewer (IK) moderated any disagreement if needed. Corresponding authors of the included studies were contacted via email for detailed information on study methodology when key criteria were determined to be *not reported* by the two reviewers.

## 3. Results

### 3.1. Literature Selection Process

The electronic database search yielded a total of 1221 results; removal of duplicates resulted in 522 remaining articles (Figure S1). Hand-searching relevant bibliographies and journals yielded one additional article [28]. Of the 523 titles/abstracts screened, a total of 21 full-text articles [7,14,15,18,19,21,22,28,29,30,31,32,33,34,35,36,37,38,39,40,41] were selected for inclusion in the review based on pre-determined eligibility criteria. Following the full-text review, five [28,37,38,39,40] of the 21 articles were excluded as they did not satisfy the eligibility criteria; Table S3 summarizes the excluded studies with reasons for exclusion. The remaining 16 studies [7,14,15,18,19,21,22,29,30,31,32,33,34,35,36,41] were included in the systematic review.

### 3.2. Description of Included Studies

#### General Characteristics of Included Studies

A total of 16 studies, including a total of 685 participants, examining epigenetic changes in periodontitis compared to healthy patients, were included in the systematic review [7,14,15,18,19,21,22,29,30,31,32,33,34,35,36,41]. All 16 studies examined DNA modifications (i.e., CpG methylation) as an outcome and no studies on histone modifications were identified in the search for inclusion in the review.

Three of the studies examined Aggressive Periodontitis (AgP) compared to healthy controls [7,14,18], one of the studies examined Localized Aggressive Periodontitis (LAP) compared to healthy controls [30], and the remaining twelve studies examined Chronic Periodontitis compared to healthy controls. In addition, two of the studies [19,36] examining Chronic Periodontitis, included experimental gingivitis as a second exposure group, compared to healthy controls.

An overview of the general characteristics of the included studies is presented in Table 1. A brief overview of the general characteristics is described below.

### 3.3. Study Design

One [22] of the fifteen included studies was a short-term prospective cohort study. The remaining fourteen studies were retrospective observational studies.

### 3.4. Setting and Study Population

Fourteen of the studies recruited patients from a university-based dental clinic [7,14,15,18,29,31,32,33,34,35,41], one of the studies recruited from the local county health department and university [30], and one study recruited from a private dental clinic [22], and three of the studies did not report the study setting [19,21,36]. Seven of the studies were conducted in Brazil [14,15,18,32,33,34,35], four in the United States [19,21,30,36], one of the studies in China [29], one in Germany [7], one in Italy [22], one in Japan [31], and one in India [41].

Further, three studies included only patients from the Southeastern region of Brazil [14,15,18], one study only patients from Central Germany [7], one study only patients from Japan, and the remaining studies did not specify the ethnic background of the included patients. Three of the studies included only Caucasians [7,18,22], one of the studies included only African Americans [30], and the remaining studies did not specify the race of the study population. Nine studies did not report the ethnic background or the race of the included study population [19,21,29,32,33,34,35,36,41].

#### Assessed Methylated Gene Sites

The methylation sites assessed are listed by study group in Table 2 and by gene site in Table 3. Key findings were reported as changes in *CCL25* [7], *PTGS2* [22], *FADD* [30], *IFNG* [19,22,35], *IL6* [31,33], *IL6R* [30], *CXCL8* [18], *IL10* [35], *IL17C* [7], *IRAK1BP1* [30], *MAP3K7* [30], *MMP9* [29], *MYD88* [30], *PPARA* [30], *PTGS2* [21], *RIPK2* [30], *SOCS1* [14,15], *SOCS3* [15], *TIMP1* [29], *TLR2* [34], and *TNF* [22,36,41] gene sites.

### 3.5. Methods for Detecting DNA Methylation Changes

Bisulfate restriction analysis combined with methylation-specific high-resolution melting analysis [15], bisulfite modification and methylation specific PCR [41], bisulfate-specific PCR, cloning, and sequencing [21], bisulfate-specific PCR and pyrosequencing [36], combined bisulfate restriction analysis (COBRA) [14], direct genome sequencing [31], high-throughput DNA methylation analysis [32], methylation-specific polymerase chain reaction (MSP) [18,33,35], PCR array [7], pyrosequencing [19,22,29,30], and methyl profiler DNA methylation qPCR assay [34] were applied as methods for detecting epigenetic changes.

### 3.6. Characteristics of The Outcomes Measured

Table 2 summarizes the study outcomes reported by the studies, including the main site-specific methylation level findings, as well as additional observations/outcomes assessed. Key findings of individual studies, as well as inter-study observations are reported below.

#### 3.6.1. Individual Study Outcomes

1. DNA methylation of candidate genes

(a) Chronic periodontitis compared to healthy subjects

Zhang et al. (2010) evaluated the DNA methylation status of the *PTGS2* promotor, with effects on *PTGS2* mRNA expression, of gingival biopsies from patients with CP compared to periodontally healthy controls [21]. The authors found a statistically significant hypermethylation of the *PTGS2* promotor, associated with lower levels of *PTGS2* expression, in CP compared to the controls [21].

Viana et al. (2011) evaluated the methylation status of *IFNG* and *IL10* in gingival tissue from subjects with CP compared to subjects without periodontitis. The authors found similar methylation profiles of both genes in both study groups and concluded that methylation is a usual feature of both genes in the diseased periodontal tissue [35].

De Faria Amormino et al. (2013) evaluated the methylation status and expression of *TLR2* in gingival samples from individuals with and without CP. The authors observed a hypermethylation profile and decreased gene expression in the CP group compared to the control group [34].

Stefani et al. (2013) assessed DNA methylation profiles and expression of *IL6* in gingival biopsy samples from patients with CP compared to controls. The authors observed no difference between the two groups in methylation profiles from two different regions of *IL6*, but higher expression of *IL6* in the CP group compared to control group, suggesting that mechanisms not associated with methylation are involved in *IL6* regulation in CP [33].

Andia et al. (2015) examined the DNA methylation patterns of *SOCS1* and *SOCS3* in epithelia and connective gingival tissue samples collected from patients with a history CP after controlling for inflammation compared to healthy patients. The authors observed no difference between study groups and tissues in *SOCS1* and *SOCS3* methylation [15].

Kobayashi et al. (2016) evaluated the DNA methylation profiles and mRNA expression of *IL6* from gingival tissue and peripheral blood in patients with CP compared to healthy controls. The authors showed that overall methylation rates were similar in the CP and healthy groups; no significant difference was observed in the methylation rates at any of the sites for gingival tissue compared to peripheral blood [31].

Asa’ad et al. (2017) analyzed DNA methylation for the putative inflammation-associated genes *PTGS2*, *IFNG*, and *TNF* in gingival biopsies from patients diagnoses with CP at baseline, and 2 and 8 weeks following periodontal therapy, compared to clinically healthy patients [22]. The authors reported that, while periodontal therapy did not influence *IFNG* or *TNF* methylation in either study group over time, periodontal therapy significantly reduced *PTGS2* methylation levels at 2- and 8-weeks post-treatment, such that methylation levels in the CP group were comparable to the healthy group.

Li et al. (2018) investigated the promotor DNA methylation status of *MMP9* and *TIMP1* in GCF from patients with CP compared to controls [29]. The authors found a positive correlation between methylation levels of *MMP9* and the severity of CP, as well as the duration of CP. The authors further identified a sex dimorphism in *MMP9* methylation in the susceptibility of CP.

Lavu et al. (2019) evaluated the methylation status of *TNF* from peripheral blood in patients with CP compared to healthy controls [41]. They found lower methylation levels in CP compared to healthy controls.

(b) Aggressive Periodontitis Compared to Healthy Subjects

Andia et al. (2010) observed the DNA methylation status of the *CXCL8* promotor of oral epithelial cells in subjects who presented with generalized AgP compared to healthy controls. The authors reported a marked hypomethylation of *CXCL8* in AgP compared to healthy subjects [18].

Baptista et al. (2014) verified the DNA methylation patterns of *SOCS1* promotor region in oral epithelium cells from AgP patients compared to controls. The authors reported that *SOCS1* was predominantly demethylated in both groups with a higher percentage of demethylation in the control group, compared to AgP group [14].

Schulz et al. (2016) quantified methylation patterns of 22 inflammatory candidate genes in gingival biopsies from patients with AgP compared to controls. The authors reported that CpG methylation of *CCL25* and *IL17C* was significantly reduced in AgP compared to periodontally healthy tissue [7].

Shaddox et al. (2017) examined the role of DNA methylation of TLR genes *FADD*, *IL6R*, *IRAK1BP1*, *MAP3K7*, *MYD88*, *PPARA*, and *RIPK2* in peripheral blood collected from patients with LAP compared to healthy controls. The authors observed significant differences in methylation between LAP patients compared to healthy controls, and further between the severe and moderate LAP; more specifically, moderate LAP patients presented hypermethylation of both the upregulating (*MAP3K7*, *MYD88*, *IL6R*, and *RIPK2*) and downregulating (*FADD*, *IRAK*, and *PPARA*) genes, while severe LAP patients presented hypomethylation of *FADD*, *IL6R*, *IRAK1BP1*, *MAP3K7*, *MYD88*, *PPARA*, and *RIPK2*. The significant differences in methylation status also correlated with an increased pro-inflammatory cytokine profile for LAP patients [30].

Chronic periodontitis vs. healthy patients against experimentally induced gingivitis Zhang’s group (2010) determined DNA methylation levels within the *IFNG* promotor in gingival biopsy samples from subjects with CP compared to experimentally induced gingivitis and healthy controls. The methylation levels of all sites analyzed within the *IFNG* promoter region were significantly lower in the CP samples compared to gingivitis and healthy control samples. *IFNG* promoter hypomethylation was also related to increased *IFNG* transcription in CP [19].

Zhang’s group (2013) investigated alterations in DNA methylation of the *TNF* promotor in gingival biopsies from CP, experimentally induced gingivitis, and healthy controls. The authors demonstrated that the *TNF* promotor was hypermethylated in CP compared to controls, but was not modified at either the induction or resolution phase of experimental gingivitis [36].

2. Genome Wide Methylation

Only two studies described genome wide methylation analyses [32,36]. De Souza et al. (2014) employed a high-throughput assay to investigate DNA methylation in gingival samples from CP and control groups, after matching individuals by age [32]. Three groups of genes were studied: Immune-inflammatory process, cell-cycle control, and stably expressed genes. Zhang et al. (2013) treated T-helper cells with a global DNA methylation inhibitor, 5-azacytidine and 5-aza-2’- deoxycytidine (5-Aza-2dC), in order to investigate the transcription level of the *TNF* promoter, which blocks global DNA methylation during replication [36]; no difference in *TNF* promoter methylation was observed.

#### 3.6.2. Results of Primary Outcomes

1. DNA methylation of candidate genes

(a) Methylation of candidate genes in periodontitis

*IL6* was assessed as a candidate gene in studies conducted by Kobayashi (2016) and Stefani (2013) [31,33]. Both groups reported no statistically significant difference in *IL6* methylation between CP and controls [31,33].

*IL6R* was assessed as a candidate gene in two of the included studies [7,30]. Schulz (2016) reported no statistically significant difference in *IL6R* methylation between AgP and controls [7]. In contrast, in the study of LAP, Shaddox (2017) observed a statistically significant increase in *IL6R* methylation in moderate LAP and severe LAP compared to healthy controls [30].

*IFNG* was assessed as a candidate gene in three of the included studies [19,22,35]. Asa’ad (2017) and Viana (2011) both reported no statistically significant difference in *IFNG* methylation between CP and controls [22,35]. In contrast, Zhang (2010) reported a statistically significant hypomethylation in CP compared to control samples [21]. Following the periodontal treatment of CP, Asa’ad’s group reported that the therapy did not influence *IFNG* methylation [22].

*PTGS2* was assessed as a candidate gene by Asa’ad (2017) and Zhang (2010). Both groups reported a statistically significant hypermethylation of *PTGS2* in CP compared to controls [21,22].

*SOCS1* was assessed as a candidate gene in two of the included studies [14,15]. Andia (2015) observed low levels of methylation overall and no statistically significant difference between CP and controls [15]. In the study of AgP, Baptista (2014) reported a statistically significant difference in *SOCS1* methylation between AgP and control groups, with a higher percentage of demethylation in the control group [14].

*TNF* was assessed as a candidate gene in three of the included studies [22,36,41]. Asa’ad (2017) reported a statically significant difference in *TNF* methylation with more methylation in the controls [22]. Similarly, Lavu (2019) found higher methylation levels of *TNF* in controls compared to CP [41]. In contrast, Zhang (2013) reported a statistically significant hypermethylation of the *TNF* promoter in CP compared to controls [36].

(b) Methylation of Candidate Genes in Gingivitis

Two of the included studies implemented an experimental gingivitis model [19,36]. Zhang (2010) examined *IFNG* as a candidate gene in experimental gingivitis samples compared to CP and healthy controls samples; the group reported a statistically significant hypomethylation in experimental gingivitis and CP samples, compared to control samples [19]. Zhang (2013) examined *TNF* methylation over time; no statistically significant difference in *TNF* methylation patterns were observed in the experimental gingivitis group between the induction and resolution phases [36].

2. Genome-Wide Methylation

De Souza’s group (2014) performed array-based methylation analysis on gingival samples from 12 periodontal cases and 11 age-matched healthy individuals [32]. The group compared microarray-based variation in DNA methylation from periodontitis and healthy control groups from 1284 immune-related gene, 1038 cell cycle-related genes, and 575 genes stably expressed under various physiological conditions. Based on 59,999 probes included in the analysis, the highest variations in DNA methylation between the periodontitis and healthy control groups were found in the immune gene group; statistically significant differences were found in 5422 immune, 4583 cell cycle, and 2349 stable genes. Mean methylation scores and frequency of methylated probes were significantly lower in genes related to the immune process in the periodontitis group compared to the healthy controls, and DNA methylation variation correlated with mRNA variations.

#### 3.6.3. Results of Secondary Outcome

1. Gene Expression

Six of the included studies [19,21,32,33,34,36] reported on transcriptomic outcomes associated with the epigenetic differences between CP and healthy study groups.

The majority of these studies [19,21,32,34,36] found an inverse association between methylation and transcription levels between the CP and healthy control groups. Zhang’s group (2010) found an inverse association between *PTGS2* methylation and *PTGS2* expression with higher methylation levels and lower gene expression in the CP group compared to healthy controls [21]. De Faria’s group (2013) reported a hypermethylated *TLR2* profile in the CP group compared to controls and higher *TLR2* transcription in the control group; no statistically significant difference was found in *TLR2* transcript levels and the number of inflammatory cells in either group [34]. De Souza’s group (2014) found a significantly lower methylation and higher gene expression of the immune group genes in the CP group compared to healthy controls; the inverse relationships between methylation and transcription were also observed for the cell cycle gene group [32]. Zhang’s group (2010) found lower *IFNG* methylation levels and significantly higher transcription in CP compared to controls [19]. Zhang’s group (2013) found increased *TNF* promotor methylation levels (CpG sites at -163 bp and -161 bp) in CP compared to controls and a statistically significant inverse association *TNF* expression at the -163 CpG site [36].

Two of the included studies [19,33] observed significant differences in transcription, but not methylation levels, suggesting that other mechanisms may be involved in the regulation of those genes [33]. Stefani’s group (2013) reported no statistically significant differences in *IL6* methylation between the CP group and controls, but a statistically significant difference in transcription between the groups [33]. Similarly, for the study of *IFNG* in experimental gingivitis, Zhang’s group (2010) found no significant differences in *IFNG* methylation between experimental gingivitis and control groups, but increased mRNA levels in the experimental gingivitis group [19].

2. Specific gene methylation after periodontal therapy

Three of the included studies reported methylation outcomes for the experimental group over-time following treatment [15,22,36] (Table 4). Andia et al. (2015) reported no differences in *SOCS1* and *SOCS3* methylation between healthy patients and controlled CP patients 3-months following periodontal therapy [15]. Asa’ad et al. (2017) reported that *TNF* and *IFNG* methylation remained stable two weeks following periodontal therapy [22]. In addition, Asa’ad et al. found significantly reduced *PTGS2* methylation levels compared to healthy controls at both 2- and 8-week follow-up periods. Finally, Zhang et al. (2013) found no significant difference in the methylation and *TNF* gene expression between the induction and resolution phases in experimentally induced gingivitis [36].

3. Association between epigenetics and clinical parameters

A total of 5 studies reported on associations between epigenetic changes and clinical parameters [29,31,33,34,35].

Two studies investigated the association between epigenetics and the severity of the periodontal disease [33,35]. Viana et al. did not find an association between the severity of periodontal disease and *IFNG* methylation in CP (data not shown) [35]. Similarly, Stefani et al. found no association between the severity of periodontal disease and *IL6* methylation in the periodontitis group [33].

Three studies investigated the association between epigenetic changes and various clinical parameters, including PD, BOP and CAL [29,31,34]. De Faria et al. found a correlation between the *TLR2* methylation and PD, but not CAL [34]. Kobayashi et al. observed a significant negative correlation in the CP group between the overall *IL6* methylation and PD, but not BOP and CAL [31]. Li et al. (2018) found a slight positive correlation between *TIMP1* promoter methylation and PD and a significantly negative correlation between *TIMP1* promoter methylation and BOP; no association between methylation levels of *MMP9* and PD, CAL, and BOP outcomes was observed [29].

#### 3.6.4. Study Quality

Quality assessment of all included studies indicated that three studies [22] [30,41] were of *good quality*, eleven of the studies [7,14,15,18,19,29,30,31,32,34,35] were of *fair* quality, and none of the included studies was assessed as poor (Table S4). A total of 5 quality criteria (items #1, #3, #7, #9, and #10) were reported by all included studies. A number of criteria were rarely reported (including items #6 and #8). Two criteria (items #5 and #12) were not reported by any of the included studies.

## 4. Discussion

### 4.1. Healthy Subjects with Periodontitis

Periodontal disease is complex and multifactorial in nature, suggesting that epigenetic events might participate in the development of the disease and in the determination of its phenotype [42]. Therefore, the evaluation of these events could be important for understanding the regulation of cytokine expression during inflammation [35]. Findings from our systematic review, presented below, suggest that epigenetic regulation of several inflammatory cytokines may impact periodontitis.

IL6 is a multifunctional pro-inflammatory cytokine that is highly expressed in patients with periodontitis [43]. However, inconsistent results were reported on its DNA methylation profile in CP. Stefani et al. (2013) noted increased methylation levels of the *IL6* promoter, which were not associated with the increased expression of IL6 in patients with CP, probably due to the presence of distinctly active mixed cell populations [33]. On the other hand, Kobayashi et al. (2016) reported hypomethylation of the *IL6* promoter in gingival tissues from CP, probably due to chronic exposure of sulcular and junctional epithelium to bacteria and inflammatory cytokines, suggesting that increased *IL6* transcription might be correlated to hypomethylation [31]. However, Kobayashi et al. (2016) reported the same levels of *IL6* transcription in CP and health [31], which is contradictory to previous studies, in which *IL6* levels were increased in periodontitis [43,44]. This can be attributed to the fact that the patients enrolled in the study by Kobayashi et al. (2016) exhibited mild periodontitis [31].

IL10 is an anti-inflammatory cytokine, which plays a vital role in periodontal diseases; polymorphisms in the *IL10* gene have been associated with periodontitis [45]. Although no differences in *IL10* methylation were observed between CP and healthy individuals [35], suggesting that hypermethylation of *IL10* promoter is a common feature in health and disease, the authors still reported the occurrence of partial methylation in the *IL10* gene. This finding might be explained by the presence of various inflammatory cells in distinct phases of activation [35].

CXCL8 is an important chemokine [46] that plays an important role in periodontal disease, since it recruits and activates acute inflammatory cells [47]. From an epigenetic point of view, *CXCL8* was markedly hypomethylated in aggressive periodontitis patients, suggesting CXCL8 as a potential etiological factor in the pathogenesis of AgP [18].

TNFα is a primary inflammatory cytokine that is elevated in active and progressive periodontitis [42,48,49]. From an epigenetic point of view, Zhang et al. (2013) reported increased *TNF* methylation in CP [36], which results in decreased expression of TNFα in the disease. As such, hypermethylation of *TNF* in CP could be a regulatory dampening mechanism that protects the host from prolonged environmental stimuli [36]. Conversely, Lavu et al. (2019) reported a lower level of *TNF* promoter methylation in the periodontitis group compared to controls [41]. The discrepancy in the findings between both studies may be attributed to the different *TNF* regions examined in each study [21,41].

IFNG is an immune regulatory cytokine that plays an important role in the progression of inflammation [50]. With regard to periodontal disease, it has been found elevated in inflamed gingival tissues, and has been associated with advanced periodontal disease and disease progression [48,51,52]. Different results were reported with respect to *IFNG* DNA methylation levels between healthy and CP subjects. While Zhang and Crivello’s group (2010) [19] found low DNA methylation in periodontitis subjects, Viana and colleagues (2011) [35] demonstrated no significant difference between groups. However, it must be noted that in Zhang’s study (2010), the magnitude of the association between hypomethylated *IFNG* promoter and CP was not significant [19].

PTGS2 is the enzyme that synthesizes prostaglandin E2 (PGE2), which plays a role in periodontal inflammation and alveolar bone destruction [53]. Zhang, Barros et al. (2010a) reported a hypermethylated promoter region of *PTGS2* in tissues affected by periodontal disease, which was associated with lower levels of *PTGS2* transcription. This might suggest an intrinsic protective mechanism that prevents further breakdown of periodontal tissues [21]. In concordance, previous studies reported downregulation of COX-2 in periodontal disease. For example, an epidemiological study revealed a negative correlation between the periodontal attachment level and PGE2 levels, in the gingival crevicular fluid [54], suggesting that chronic inflammation might serve as a new “set-point,” in which specific inflammatory mediators are downregulated to restrict further tissue destruction [21].

Toll-like receptors (TLRs) play an important role in the inflammatory process in periodontitis [55].

De Faria Amormino et al. (2013) reported hypermethylation of *TLR2* in CP patients compared to healthy controls [34], suggesting that *TLR2* hypermethylation might play a role in the development of periodontitis. The authors reported that TLR2 hypermethylation in CP was associated with low transcription of the gene. Although this finding contradicts previous reports on increased mRNA levels of *TLR2* in gingival tissues from periodontitis patients [56,57], the authors suggested that the low expression of TLR2 reported in their study could result in the reduction of inflammatory mediators and encourage the chronic persistence of bacteria in the periodontium and subsequent development of periodontitis [34].

Shaddox et al. (2017) assessed DNA methylation in localized aggressive periodontitis (LAP) for TLR signaling pathway genes, that either result in the upregulation (*MYD88*, *MAP3K7*, *RIPK2*, *IL6R*) or downregulation (*FADD*, *PPARA*, *IRAK1BP1*) of TLR-mediated inflammation [30]. They observed that subjects with moderate LAP had hypermethylation of both downregulating and upregulating genes, suggesting a regulatory mechanism that could prevent further tissue destruction. Conversely, subjects with severe LAP showed a trend towards hypomethylation of upregulating genes when compared to healthy controls and subjects with moderate LAP, suggesting increased expression of pro-inflammatory cytokines that promote further periodontal tissue destruction.

MMP9 and TIMP1 were also investigated as inflammatory mediators. Abnormal protein expression of MMP family was previously reported in periodontitis and TIMP1, an endogenous inhibitor of MMPs [29] was involved in tissue invasion in periodontitis [58]. Li et al. (2018) reported significantly higher methylation of the MMP9 gene promoter in periodontitis patients, compared to healthy subjects [29]. Methylation levels of the MMP-9 promoter were also associated with the duration of periodontitis. These findings suggest that reduced expression of MMP9, due to its methylation, might play a role in the pathogenesis of periodontitis [29]. However, this pattern of expression is contradictory to what has been published, since MMP9 is the most abundant in tissues affected by periodontal disease [59]. Other studies confirmed elevated levels of MMP9 in GCF and gingival tissue of periodontitis patients [60,61]. Interestingly, Li et al. (2018) reported that MMP9 methylation levels differed by sex and age, suggesting the possibility for a gender-specific biomarker in monitoring the risk of periodontitis [29]. Next, in regard to TIMP1, the high methylation levels of *TIMP1* in severe periodontitis indicate its reduced expression at this stage of the disease. This is in concordance to previous findings in which active sites tended to decrease TIMP1 levels during the progression of CP [62].

Another mediator that was assessed for its DNA methylation profile is *SOCS1*, one of the most potent inhibitors of cytokine signaling [63]. *SOCS1* showed more hypomethylation in healthy subjects compared to those with aggressive periodontitis [14], Findings from *SOCS1* are interesting because it is a gene that controls inflammation, and thus, its methylation pattern might participate in the development of aggressive periodontitis.

Inflammatory mediators IL17C and CCL25 play a fundamental role in T-cell development and regulate innate epithelial immune responses, respectively [7]. Schulz et al. (2016) analyzed DNA methylation levels of 22 inflammatory genes from gingival tissues of patients with aggressive periodontitis and healthy subjects, and observed lower methylation of *IL17C* and *CCL25* in periodontitis patients [7]. The decrease in CpG methylation is presumably accompanied by an increase in gene expression. This could lead to greater availability of IL17C CCL25 and subsequent loss of the periodontal attachment. Consistent with these findings, previous studies have shown an upregulation of IL-17 in periodontitis patients compared to healthy tissues [64].

In one of the earliest published studies on epigenetic regulation in periodontal disease, DNA methylation levels were assessed in immune cell and cell cycle genes [32]. Higher variations in DNA methylation between CP and controls were observed in genes related to the immune-inflammatory process, which might suggest that modulation of mRNA transcription of the immune-inflammatory gene might influence the prognosis of CP [32].

Review of the published literature on epigenetic changes in periodontitis suggests that methylation of certain periodontitis-related genes might either further contribute to or limit the destruction of periodontal tissues. In some studies, the methylation profile confirmed the expression levels of cytokines/mediators that were reported in classical studies, while other studies showed inconsistencies. Therefore, functional studies are needed to better elucidate the role of epigenetics in periodontitis pathogenesis.

### 4.2. Effect of Periodontal Therapy of Periodontitis on DNA Methylation

The prospective positive effects of periodontal therapy on the methylation profile of DNA and specific genes was first reported by Andia et al. (2015); the group observed no change in DNA methylation of *SOCS1* and *SOCS3* between healthy and periodontitis tissues three months following periodontal therapy [15]. However, variations in the methylation levels between both groups were not assessed at baseline and samples of inflamed tissues were not investigated [15].

Subsequently, Asa’ad et al. (2017) monitored the changes in DNA methylation of *PTGS2*, *IFNG*, and *TNF* inflammatory genes in periodontitis patients at baseline and following periodontal therapy in comparison with healthy subjects; outcomes were assessed at both the site and patient levels [22]. The group showed that periodontal therapy was correlated with alterations of *PTGS2* gene methylation in patients with CP, while DNA methylation levels of *TNF* and *IFNG* remained unchanged in the periodontitis group after periodontal therapy. Moreover, the DNA methylation status of the *TNF* gene promoter was almost stable in normal sites in periodontitis patients throughout the evaluation period and was not restored after periodontal therapy to that of healthy patients. This could indicate a different methylation profile in subjects with and without a history of periodontitis. Overall, findings from Asa’ad’s study suggest that local epigenetics changes, which may be modulated by environmental factors, including microbiota, may have an effect on periodontal tissue [22].

### 4.3. Study Limitations

The present systematic review was not able to establish a difference in DNA methylation patterns between methods for sample collection, due to a high heterogeneity (i.e., biopsies, GCF, blood, epithelial buccal cells) and inconsistency of CpG sites assessed. Therefore, caution is warranted during the interpretation of the results.

### 4.4. Future Research

Clearly, further clinical studies are needed to evaluate DNA methylation changes in relation to periodontal disease and its treatment in human adults. Future investigations should further aim to identify specific factors that affect the local epigenetic changes in periodontal soft and hard tissues, such as epidrugs that can be immobilized on membranes, in order to locally improve bone formation and/or reduce inflammation. Moreover, future studies should comprehensively assess DNA methylation. Finally, future studies should improve the reproducibility of their findings by better controlling: (1) Heterogeneity of the study population, as racial and ethnic differences in methylation, have been established, and none of the included studies accounted for this source of heterogeneity; (2) independent replication; (3) more consistent approach to methylation analysis, i.e., the same sites need to be evaluated; and (4) making data publicly available in order to allow for meta-analysis of study findings.

## 5. Conclusions

Due to the limited number of studies and heterogeneity in study design and outcomes, there is insufficient evidence to support or refute the association between DNA methylation and periodontal disease and its treatment in human adults. However, several included studies found associations between methylation levels and periodontal disease risk. Further research must be conducted to identify reproducible epigenetic markers and determine the extent to which DNA methylation can be applied as a clinical biomarker.

## 6. Clinical Relevance

### 6.1. Scientific Background

Despite the number of reports in the literature on the role of epigenetic mechanisms in periodontal disease, a thorough assessment of the published studies is still needed, to better comprehend the evidence on the relationship between epigenetic changes and periodontal disease, and its treatment.

### 6.2. Principal Findings

There was some evidence, albeit inconsistent, for an association between DNA methylation and periodontal disease.

### 6.3. Practical Implications

In light of the associations between the methylation levels of selected genes and periodontal disease, it seems that epigenetic markers may be applied in the future for chairside periodontal disease risk stratification and monitoring treatment outcomes.

## Figures and Tables

**Table 1:** Characteristics of the included studies.

Study Characteristics							
Study/Year/Reference	Design/Study Period	Setting, Location, Funding, and COI	N Patients—Sex	Mean Age in Years (Range/SD)	Racial/Ethnic Background	Exclusion Criteria	Smokers
Healthy Controls vs. Periodontitis							
Andia et al., 2010 [18]	Observational 2008–2009	University, Brazil Founding: public COI: none	1. AgP: 37 28F/9M 2. Controls: 37 28F/9M	1. AgP: 28.81 ± 4.73 2. Controls: 28.08 ± 6.39	Southeastern region of Brazil; only whites	Excluded: Medical history (DM, hepatitis or HIV infection), a genetic disease with periodontal manifestations, diseases of oral hard/soft tissues. (other than caries/periodontal disease), pre-medication for dental treatment, chronic usage of corticosteroids, immunosuppressors, ANUG	Excluded: All smokers
Zhang et al., 2010 [21]	Observational NR	Location of recruitment: NR; approved by the IRB of the University of North Carolina at Chapel Hill, USA Funding: university COI: NR	1. CP: 10 5F/5M 2. Controls: 6 5F/1M	1. CP: 45.8 ± 7.4 2. Controls: 44.2 ± 15.6	NR	Excluded: Abs or NSAIDs within one month of surgery; treatment for other diseases received within three months	NR
Viana et al., 2011 [35]	Observational 2008–2009	University, Brazil Founding: public COI: NR	1. CP: 18 4F/12M 2. Controls: 16 5F/13M	1. CP: 50 (26–60) 2. Control: 29.5 (19–53)	NR	Excluded: Systemic disorders that could influence the course of periodontal disease, chronic usage of anti-inflammatory drugs or other drug therapy, severely compromised immune function	Excluded: All smokers
de Faria Amormino et al., 2013 [34]	Observational 2011–2011	University, Brazil Funding: non-profit foundation COI: NR	1. CP: 20 10F/10M 2. Controls: 20 10F/10M	1. CP: 36.75 ± 7.79 2. Controls: 36.75 ± 7.79	NR	Excluded: Orthodontic appliances, anti-inflammatory drugs, history of diabetes, hepatitis or HIV infection, immunosuppressive chemotherapy, bleeding disorders, severely compromised immune function, systemic antibiotics within six months	Excluded: All smokers
Stefani et al., 2013 [33]	Observational NR	University, Brazil Funding: public and non-profit foundation COI: NR	1. CP: 21 15F/6M 2. Controls: 21 13F/8M	1. CP: 41 (25–58) 2. Controls: 28 (18–69)	NR	Excluded: Systemic disorders that could influence the course of periodontal diseases, chronic anti-inflammatory drugs, severely compromised immune function	Excluded: All smokers
Baptista et al., 2014 [14]	Observational 2011–2012	University, Brazil Funding: public foundation COI: None.	1. AgP: 30 24F/6M 2. Controls: 30 20F/10M	1. AgP: 29.20 ± 5.60 2. Controls: 37.23 ± 12.82	Southeastern region of Brazil	Excluded: Oral hard/soft tissues diseases (except caries and periodontitis), orthodontic appliances, pre-medication for dental treatment or chronic use of anti-inflammatory drugs, DM, hepatitis, HIV infection, immunosuppressive chemotherapy, history of severely compromising immune function, ANUG	Excluded: All smokers
De Souza et al., 2014 [32]	Observational NR	University, Brazil Funding: public foundation COI: None.	1. CP: 12 7F/5M 2. Controls: 11 6F/5M	1. CP: 50.63 ± 7.89 2. Controls: 50.42 ± 8.35	NR	Excluded: systemic disorders that could affect the periodontal condition; antibiotics and anti-inflammatory medication within the past six months	Excluded: All smokers
Andia et al., 2015 [15]	Observational 2010–2012	University, Brazil Funding: public foundation COI: none l	1. CP: 10 6F/4M 2. Controls: 10 7F/3M	1. CP: 53.9 ± 9.9 2. Controls 48.3 ± 8.9	Southeastern region of Brazil	Excluded: Oral hard/soft tissue diseases (except caries and periodontitis), orthodontic appliances, pre-medication for dental treatment, chronic use of anti-inflammatory drugs, DM, Hep., HIV, immunosuppressive chemotherapy, or history of any other disease known to severely compromise immune function, ANUG, or pregnancy/lactation.	Excluded: All smokers
Kobayashi et al., 2016 [31]	Observational 2013–2014	University, Japan Funding: public COI: none	1. CP: 25 14F/11M 2. Controls: 20 12F/8M	1.CP: 64.3 ± 1.4 2. Controls: 65.4 ± 2.2	Japanese	Excluded: Diabetes mellitus, pregnancy, fewer than 15 teeth, and history of any periodontal therapy or medication within the previous three months	Included: Current-smokers, former-smokers, or never-smokers *All never-smokers except one former-smoker in the CP group
Schulz et al., 2016 [7]	Observational NR	University, Germany Funding: non-profit foundation and university COI: none	1. AgP: 15 7F/8M 2. Controls: 10 6F/8M	1. AgP: 41.4 ± 10.5 2. Controls: 36.9 ± 17.5	Caucasians of Central Germany	Excluded: Drug-induced gingival hyperplasia, antibiotics in the last six months, chronically used anti-inflammatory drugs or had a history of inflammatory diseases of the oral cavity (including herpes simplex infections) or diseases associated with periodontitis	NR
Asa’ad et al., 2017 [22]	Short-term prospective cohort study 2015–2016	Private dental practice, Italy Founding: self-supported. COI: none	1. CP: 10 7F/3M 2. Controls: 10 5F/5M	1. CP: 46.6 (26-60) ± 10 2. Controls 53.3 (25-69) ± 12.3	Caucasians	Excluded: Antibiotics and/or NSAIDs, for at least one month before enrollment; periodontal therapy within the last three months prior to enrollment	Included: Non-smokers or ex-smokers who had quit smoking for at least one or more years prior to enrollment in the study
Shaddox et al., 2017 [30]	Cross-sectional 2007–2014	Governmental and university, USA Funding: public COI: none	1. LAP: 20 (severe:10, moderate:10) Severe: 9F/1M; Moderate: 5F/5M 2. Controls: 20 12F/8M	1.LAP: Severe: 13.70 ± 3.74 Moderate: 17.00 ± 2.21 2. Controls: 14.53 ± 5.39	African-American race	Excluded: Systemic diseases or conditions that influence the progression/clinical characteristics of periodontal disease, antibiotics within last three months, smoker, taking any medications that could affect the LAP, pregnant/lactating.	Excluded: All smokers
Li et al., 2018 [29]	Observational 2016–2017	University, China Funding: public COI: none	1. CP: 88 (severe:27, moderate:29, mild:32) 52F/36M 2. Controls: 15 NR	1. CP: 35.25 (25–62) 2. Control: 25.5 (range/SD: NR)	NR	Excluded: Systemic diseases (such as cancer, coronary heart disease, diabetes, hepatitis, or blood diseases); smoking; pregnant/lactating; AgP; antimicrobial/anti-inflammatory/periodontal treatment therapy within the last six months; environmental poison exposure	Excluded: All smokers
Lavu et al., 2019 [41]	Case-control NR	University, India Funding: NR COI: NR	1. CP: 25 11F/14M 2. Control: 25 16F/9M	1. CP: 36.45 ± 5.45 2. Control: 32.44 ± 3.20	NR	Excluded: Smoking, antibiotics within last six months, analgesics within one week, pregnant/lactating, systemic disease, previous periodontal treatment	Excluded: All smokers
**Other periodontal disease comparison: experimental gingivitis**							
Zhang et al., 2010 [19]	Observational NR	Location of recruitment/observational study NR; approved by the IRB of the University of North Carolina, Chapel Hill, North Caroline, USA Funding: public COI: none	1. CP: 12 3F/9M 2. Experimental gingivitis: 12 7F/5M 3. Controls: 23 17F/6M F/M CP vs. controls *p* < 0.05	1. CP: 47.2 (19–63) ± 7.4 2. Experimental gingivitis 35.8 ± 11.2 3. Controls: 40.8 ± 11.6	NR	Excluded: Antibiotics or NSAIDs within one month; medical treatment for other diseases within three months	NR
Zhang et al., 2013 [36]	Cross-sectional 2007–2009	Location of recruitment/observational study NR; approved by the IRB of the University of North Carolina, Chapel Hill, North Caroline, USA Funding: public COI: none	1. CP 17 7F/11M 2. Experimental gingivitis: 11 6F/5M 3. Controls: 18 12F/5M	1. CP 48.7 ± 8.7 2. Experimental gingivitis: 36.8 ± 9.7 3. Controls: 40.9 ± 13.5	NR	Excluded: Antibiotics or NSAIDs within one month; medical treatment for systemic diseases within three months	NR

**Table 2:** Description of epigenetic testing and findings.

Study/Year/Reference	Methodology						Results/Conclusions				
	N Cases per Study Group	Criteria for Subject Allocation	Adjustment (e.g., Age, Sex, Smoking, etc.)	Type of Tissue (and Site Collected, If Applicable)	Methylation Sites (Genes) Assessed	Methods for Detecting DNA Methylation Changes	Main Findings	Additional Observations/Outcomes Assessed	Global/Genome-Wide Reporting	Association between DNA Methylation and Clinical Parameters	Clinical Parameters
**Healthy Controls vs. Periodontitis**											
Andia et al., 2010 [18]	1. AgP: 37 2. Controls: 37	1. AgP: AAP classification, 1999. 2. Controls: Absence CAL and no sites with PD ≥ 3 mm.	NR	Buccal Epithelial Cells	*CXCL8*	MSP	***CXCL8*:** Hypomethylated in AgP vs. controls	Methylation status is not the same in different tissues or in all cells of the same tissue; thus, the different cells in GT, such may promote inconclusive results. Future confirmatory studies using laser capture microdissection to isolate gingival epithelium.	NR	NR	1.AgP: PD: 6.9–0.6 mm 2.Comtrol: NR
Zhang et al., 2010 [21]	1. CP: 10 2. Controls: 6	1 CP: PBs of 5 mm or more, BOP, and radiographic evidence of localized bone loss. 2. Controls: Periodontally healthy or localized mild gingivitis at non-study sites (no BOP and PD ≤ 4mm).	NR	GT: CP: PD ≥ 5mm, BOP and localized BL Control: PD ≤ 4 mm, no BOP	*PTGS2*	Bisulfite Specific PCR, Cloning, and Sequencing	***PTGS2*:** Hypermethylated in CP (5.06-fold higher) vs. controls (*p* = 0.03).	Relationships between methylation status and clinical status are tenuous and should be confirmed in larger studies. The increase in methylation in CP was associated with a metastable level of *PTGS2* mRNA expression that was lower than that seen in non-inflamed participants with shallow sites.	NR	NR	1. CP: PD: 6.2 ± 0.6 mm CAL: 3.8 ± 1.1 mm Bone loss: yes 2. Controls: PD: 2.5 ± 0.8 mm CAL: 1.6 ± 1.1 mm Bone loss: no
Viana et al., 2011 [35]	1. CP: 18 2. Controls: 16	1. CP: AAP classification, 1999. Sites classified in relation to severity: moderates with sites with PD ≤ 6 mm; and severe disease with PD > 6 mm. 2. Controls: no periodontal disease, absence CAL, no sites with PD >3 mm and absence of dental mobility.	NR	GT: CP: PD ≥ 4 mm, BOP and extensive ABL Control: PD ≤ 3 mm, no CAL, no BOP	*IFNG*, *IL10*	MSP	***IFNG*:** No significant difference between groups ***IL10***: No significant difference in between groups	Increased number of inflammatory cells in the control group, but not statistically different vs. CP. This contradiction is due to periodontal therapy received by individuals with periodontitis for ethical purposes	NR	*IFNG* and severity of CP: No association between the severity of periodontal disease and *IFNG* methylation status in CP (data not shown). Relation between the severity of periodontal disease and *IL10* genes: NR	1. CP: PD: 7 (4–9) mm CAL: 3 8 (5–10) mm FMBS0: >30% 2. Controls: PD: ≤3 mm CAL: ≤3 mm FMBS: <20%
de Faria Amormino et al., 2013 [34]	1. CP: 20 2. Controls: 20	1. CP: AAP classification, 1999. 2.Controls: no periodontal disease, no CAL, no BOP, CAL, PD ≤3 mm.	NR	GT: CP: PD ≥ 4mm, BOP and extensive ABL Control: PD ≤3 mm, no CAL, no BOP	*TLR2*	Methyl Profiler DNA Methylation qPCR assay.	***TLR2***: Hypermethylated profile in CP vs. control (*p* < 0.001).	No statistically significant difference in *TLR2* transcript levels and the number of inflammatory cells in either group. *TLR2* transcription higher in control vs. CP. No correlation with the mRNA expression level in CP (PD, *p* = 0.159, r = −0.346; CAL, *p* = 0.372, r = 0.224).	NR	*TLR2* and PD: Correlation between the *TLR2* methylation frequency and PD (*p* < 0.001; r = 0.676) *TLR2* and CAL: No correlation between CAL and TLR2 methylation (*p* = 0.544; r = −0.144).	1. CP: PD: 7.75 ± 1.92 mm CAL: 9.15 ± 2.32 mm FMBS: 64.15 ± 17.38% Bone loss: yes 2. Controls: PD: 1.58 ± 1.12 mm CAL: 2.17 ± 0.58 mm FMBS: 8.84 ± 5.44% Bone loss: no
Stefani et al., 2013 [33]	1. CP: 21 2. Controls: 21	1. CP: AAP classification, 1999. Sites classified in relation to severity: Moderates with sites with PD ≤ 6 mm; and severe disease with PD > 6 mm. 2. Controls: No periodontal disease, PD ≤3 mm.	NR	GT: CP: PD ≥ 4mm, CAL ≥ 3 mm and BOP Control: PD ≤3 mm	*IL6*	MSP	***IL6***: Majority of the samples in the control and CP had partial methylation (positive for both methylated and unmethylated sequences) in the IL-6 gene considering both MSP1 and MSP2.	Higher frequency of GG genotype was observed in severe vs. moderate CP, although it was no significance (*p* = 0.06). Expression of *IL6* was higher in CP vs. control group (*p* = 0.041). No significant difference moderate vs. severe CP.	NR	*IL6* and severity of CP: No association between the severity of periodontal disease and *IL6* methylation status in the periodontitis group (data not shown).	NR
Baptista et al., 2014 [14]	1. AgP: 30 2. Controls: 30	1. AgP: AAP classification, 1999. 2. Controls: No CAL and PD greater than or equal to 3 mm, presented (FMBS) less than or equal to 25% and absence of tooth mobility.	NR	Buccal epithelial cells	*SOCS1*	COBRA	***SOCS1*:** Predominant demethylation in both groups, although Controls had a higher percentage of demethylation vs. AgP (97.5%–control vs. 92.2%–AgP) (*p* < 0.001)	Cells collected by mouthwash allow for detecting alterations in the DNA in a non-limited manner	NR	NR	1. AgP: PD: 7.10 ± 1.30 mm CAL: 7.46 ± 1.86 mm PI: 42.70 ± 24.90 GI: 35.60 ± 17.80 2. Controls: PD: 2.00 ± 0.70 mm CAL: 2.20 ± 1.20 mm PI: 25.40 ± 4.60 GI: 11.30 ± 2.20
De Souza et al., 2014 [32]	1. CP: 12 2. Controls: 11	1. CP: More than three teeth demonstrating (CAL) ≥ 5 mm and BOP. 2. Controls: No signs of periodontitis or gingival and/or periodontal inflammatio, no BOP and all teeth with a CAL level ≤ 3.5 mm.	Age- and sex-matched	GT: CP: CAL ≥ 5 mm and BOP Control: CAL ≤ 3.5 mm	Immune cell genes, stable cell genes, cell cycle genes	High-throughput DNA methylation analysis	Variations in DNA methylation between CP and controls higher in genes related to the immune inflammatory process than cell cycle and stable gene groups. **Immune group:** A significant difference in methylation and lower methyl scores (*p* = 8.8 × 10^−14^) in CP **Cell cycle genes and stable genes:** No significant differences in methylation were found between CP group and control	In order to investigate the association between the variations in methylation and mRNA levels in the three groups, we used the differential expression dataset between healthy and periodontitis GTs from Demmer and collaborators. A significant difference in expression and methylation of immune group genes. Lower methyl scores in the immune group associated with increased gene expression; the inverse relationship between methylation and mRNA levels also in cell cycle genes.	Out of 59,999 probes analyzed for immune-inflammatory process, cell-cycle and stably expressed genes, 12,049 presented significant difference (q < 0.05) when comparing samples of normal to CP individuals. Genes of the stable group were significantly more methylated than cell cycle group. There is an inverse association between variations in methylation and mRNA levels of immune genes during periodontitis. Methylation variations in key genes of T-cell differentiation were detected.	NR	NR
Andia et al., 2015 [15]	1. CP: 10 2. Controls: 10	1. CP: AAP classification, 1999; good general healthy, older than 35 years, at least eight teeth with CAL ≥5 mm, BOP and ABL. 2. Controls: Absence of attachment loss associated with periodontal pockets, FMBS <25%, no tooth mobility, no history of periodontitis.	NR	GT (ET, CT): CP: PD ≤4 mm without inflammation but BOP-positive at baseline Control: NR	*SOCS3*, *SOCS1*	Bisulfite restriction analysis combined with Methylation Specific-High Resolution Melting Analysis	***SOCS1***, ***SOCS3*:** Three months following control of inflammation in GTs, methylation profile similar between CT and ET from patients that were previously affected or not by chronic inflammation.	Results reported three months after initial therapy.	NR	NR	1. CP: CAL: 6.1 ± 0.8 mm 2. Controls: CAL: NR (*absence of CAL required for control*)
Kobayashi et al., 2016 [31]	1. CP: 25 (mild (19), moderate (4), and severe CP (2)) 2. Controls: 20	1. CP: AAP classification, 1999 (for severity Flemmig 1999). 2. Controls: no signs of periodontitis, with the absence of sites with CAL of > 3 mm.	NR	PB GT: CP: PD ≥4 mm and BOP Control: PD ≤ 2 mm, CAL ≤ 3, BOP-negative	*IL6*	Direct sequencing of genomic DNA	***IL6***: Hypomethylated in the GT vs. PB of CP (*p* < 0.001), but not in control. However, no significant differences between CP and Control in the overall methylation for GT and PB.	*IL6* mRNA expression higher in the GT vs. PB in the CP group than in the Control group (*p* = 0.03). Similar levels of *IL6* mRNA expression in GT and PB samples of CP and control. Characterized patients by their smoking status.	NR	*IL6*, and PD, BOP and CAL: A significant negative correlation in CP between methylation rate in GT and PD (*p* = 0.003), and no differences on BOP (*p* = 0.37) and CAL (*p* = 0.37)	1. CP: PD: 2.9 ± 0.1 mm CAL: 3.3 ± 0.2 mm BOP: 17.0 ± 2.6% Plaque: 34.7 ± 4.0% 2. Controls: PD: 2.3 ± 0.1 mm CAL: 2.4 ± 0.1 mm BOP: 2.1 ± 0.7% PI: 9.7 ± 1.4%
Schulz et al., 2016 [7]	1. AgP: 15 2. Controls: 10	1. AgP: AAP classification, 1999. 2. Controls: No or localized mild periodontitis. Percentage of sites with CAL ≥ 4mm of each control was ≤30%.	NR	GT: CP: PD ≥6mm Control: CAL ≤3	*ATF2*, *CCL25*, *CXCL14*, *CXCL3*, *CXCL5*, *CXCL6*, *FADD*, *GATA3*, *IL10RA*, *IL12A*, *IL12B*, *IL13*, *IL13RA1*, *IL15*, *IL17C*, *IL17RA*, *IL4R*, *IL6R*, *IL6ST*, *IL7*, *INHA*, *TYK2*	PCR Array	***CCL25 and IL17C:*** Differences in epigenetic methylation according to periodontal status. A higher percentage of methylation in controls compared to AgP of both *CCL25* (*p* = 0.015) and *IL17C* (*p* = 0.002)	The inclusion of only 25 highly selected individuals in this pilot study provides no power for statistically sufficient analyses. For the control group development of severe periodontitis at a later time cannot be excluded. Therefore, the epigenetic dependent effect, due to this possible selection bias may likely be underestimated.	NR	NR	All periodontal data reported at the site of biopsy: 1. AgP: PD: 7.20 ± 1.6 mm CAL: 8.60 ± 2.7 mm GI: 1.33 ± 0.7 PI (median, 25^th^/75^th^ percentiles): 1.0 (0/1.0) 2. Controls: PD: 2.35 ± 0.67 mm CAL: 2.35 ± 0.67 mm GI: 0.50 ± 0.5 PI (median, 25^th^/75^th^ percentiles): 1.0 (0/1.0)
Asa’ad et al., 2017 [22]	1. CP: 10 2. Controls: 10	1. CP: AAP classification, 1999. 2. Controls: PD in all sites were ≤3 mm, without any signs of tooth mobility, BOP or CAL.	NR	GT: CP (2 sites, at baseline, 2 and 8 weeks): one healthy site: PD≤3 mm, BOP and tooth mobility negative; one periodontitis site: PD ≥5 mm and BOP, at baseline Control: PD≤3 mm, BOP and tooth mobility negative	*COX2*, *IFNG* and *TNF*	Pyrosequencing	**COX2**: Hypomethylated Control compared to periodontitis sites in CP (*p* = 0.03) **TNF**: Similar methylation between CP and control, although more methylated in control (*p* > 0.05) **IFNG**: Hypermethylated in all groups, higher in control (*p* > 0.05)	Periodontal therapy significantly reduced *COX2* methylation levels in periodontitis sites at 2 (*p* < 0.001) and 8 (*p* = 0.004) weeks after treatment, comparable to Control. Epigenetic changes need to be monitored on the long run to know if methylation status in CP could become similar to healthy individuals with no history of CP.	NR	NR	(At baseline) 1. CP: PD: 4.2 ± 0.4 mm CAL: 4.2 ± 0.9 mm BOP: 63 ± 25% 2. Controls: PD: ≤3 mm CAL: ≤3 mm BOP: 0%
Shaddox et al., 2017 [30]	1. LAP: 20 ((severe (10), moderate (10)) 2. Controls: 20	1. LAP: AAP classification, 1999. 2. Controls: < 25% BOP, no attachment loss or ABL.	Age-matched unrelated participants	PB	*FADD*, *MAP3K7*, *MYD88*, *PPARA*, *IRAK1BP1*, *RIPK2*, and *IL6R*	Pyrosequencing	**A) Upregulators** (*MYD88*, *MAP3K7*, *RIPK2*, *IL6R*) of the TLR pathway: -Moderate LAP> severe LAP; ***MAP3K7*:** (*p* < 0.01) ***RIPK2*:** (*p* < 0.001), and when compared to control (*p* < 0.001) ***IL6R*:** (*p* < 0.05), and when compared to control (*p* < 0.001) ***MYD88*:** (*p* < 0.001) **B) Downregulators** (*FADD*, *PPARA*, *IRAK1BP1*) of the TLR pathway: -Moderate LAP> severe LAP ***FADD*:** (*p* < 0.001), and when compared to control = (*p* < 0.001) ***PPARA*:** (*p* < 0.001), and when compared to control (*p* < 0.01) ***IRAK1BP1*:** (*p* < 0.001), and when compared to control (*p* < 0.001) -Patients with severe LAP: Hypomethylation at the tested sites.	Significant correlations between methylation levels and cyto/chemokine-stimulated levels. Significant positive correlations: *FADD* positions 2 and 5 and pro-inflammatory (*INFG*, *IL1B*, *IL6*, *TNF*, among others) and the anti-inflammatory cytokine (*IL10*). Methylation levels of *MYD88* in all positions and *IL6R* position 3: significant negative correlations with several of these cyto/chemo-kines	NR	NR	1a. Severe LAP: PD: 5.85 ± 0.9 mm CAL: 4.06 ± 1.24 mm BOP: 14.5 ± 9.54% Plaque: 45.60 ± 27% 1b. Moderate LAP: PD: 5.38 ± 0.8 mm CAL: 2.10 ± 0.45 mm BOP: 14.20 ± 9.39% Plaque: 36.40 ± 17.85% 2. Controls: PD: 2.74 ± 0.6 mm CAL: 0 mm BOP: 13.11 ± 9.44% Plaque: 22.84 ± 11.31%
Li et al., 2018 [29]	1. CP: 88 (severe (27), moderate (29), mild (32)) 2. Controls: 15	1. CP: AAP classification, 1999 2. Controls: PD <2 mm, no attachment loss, no BOP or ABL	NR	GCF	*MMP9* and *TIMP1* promotors/CpG islands	Pyrosequencing	***MMP9*** Methylation levels: No significant difference among groups ***TIMP1*** Methylation: Increased with periodontitis severity with highest methylation in severe periodontitis (*p* < 0.001)	Female patients vs. male: Lower methylation levels of *MMP9* but higher methylation levels of *TIMP1*, and the methylation levels of *TIMP1* gradually decreased with age.	NR	*TIMP1* and PD, BOP and CAL: slight positive correlation between *TIMP1* promoter methylation and PD, and a significantly negative correlation between *TIMP1* promoter methylation and BOP. *MMP9* and PD, CAL and BOP: No association between methylation levels of *MMP9* and PD, CAL and BOP.	1a. Severe CP: PD: 3.88 ± 0.83 mm CAL: 3.0 ± 0.38 mm BOP: 60.78 ± 17.57% 1b. Moderate CP: PD: 3.09 ± 0.58 mm CAL: 2.7 ± 0.52 mm BOP: 53.52 ± 15.91% 1c. Mild CP: PD: 2.45 ± 0.21 mm CAL: 2.50 ± 0.29 mm BOP: 41.69 ± 13.48% 2. Controls: PD: 1.52 ± 0.30 mm CAL: 0 mm BOP: 20.63 ± 3.54%
Lavu et al., 2019 [41]	1. CP: 25 2. Control: 25	1. CP: AAP classification, 1999 2. Controls: no attachment loss, no BOP, no mobility, no furcation, no history of periodontal disease	NR	PB	*TNF* (-239, -245)	Bisulfite modification and methylation specific PCR [MS-PCR]	***TNF*:** Lower level of methylation in CP vs. control. 1. CP:20% (5/25) complete methylation, 48% (12/25) partial methylation, and 32% (8/25) unmethylated. 2. Control: 8% (2/25) complete methylation, 72% (18/25) partial methylation, and 20% (5/25) un-methylated Ct value (mean): 1. CP: Methylated: 28.09, unmethylated: 25.45; 2. Control: Methylated: 24.36, unmethylated: 25.15	No additional outcomes reported.	NR	NR	1. CP: PD: 5.3 ± 0.65 mm CAL: 4.5± 0.75 mm GI: 2.35 ± 0.65 PI: 2.25 ± 0.75 2. Controls: PD: 1.7 ± 0.30 mm CAL: 0 ± 0 mm GI: 0.45 ±0.15 PI: 0.95 ± 0.20
**Periodontitis, Experimental Gingivitis and Healthy Control**											
Zhang et al., 2010 [19]	1. CP: 12 2. Experimental gingivitis: 12 3. Controls: 23	1. CP: PD ≥ 5 mm, BOP, and ABL 2.Experimental gingivitis: PD ≤ 4 mm, BOP ≥ 10%, and no ABL 3.Controls: PD ≤ 4 mm, no BOP, and no ABL	NR	GT CP: PD ≥5 mm, BOP, and ABL Experimental gingivitis: PD ≤ 4 mm, no BOP, and no ABL Control: PD ≤ 4 mm, no BOP, and no ABL	*IFNG*	Bisulfite-Specific PCR and Pyrosequencing	***IFNG*:** Significant lower level of methylation in CP vs. control (*p* = 0.007) and experimental gingivitis (*p* = 0.02); no significant difference in the experimental gingivitis vs. controls Site-specific CpG sites –54, -295, +171: Significantly lower methylation level in CP vs. control at CpG sites –54 (*p* = 0.04), -295 (*p* = 0.002), and +171 (*p* = 0.0007)	mRNA level of *IFNG*: transcriptional level of *IFNG* was 1.96-fold increased and significantly higher in CP vs. controls (*p* = 0.04), and 8.5-fold increase and was significantly higher (*p* = 0.01) than tissues with periodontal health, Increased mRNA level in experimental gingivitis vs. controls but no significant methylation difference	NR	NR	1. CP: PD: 6.3 ± 0.8 mm CAL: 4.7 ± 1.5 mm 2. Experimental gingivitis: PD: 2.24 ± 0.63 mm CAL: 1.3 ± 0.45 mm 2. Controls: PD: 2.2 ± 0.6 mm CAL: 1.2 ± 0.6 mm
Zhang et al., 2013 [36]	1. CP: 17 2. Experimental gingivitis: 11 3. Controls: 18	1. CP: PD ≥ 5 mm, CAL, and BOP 2.Experimental gingivitis: PD ≤ 4 mm, BOP ≥ 10%, and no ABL 3.Controls: PD ≤ 4 mm, no BOP, and no ABL	NR	GT CP: PD ≥ 5mm, and ABL Experimental gingivitis: PD ≤ 4 mm, no BOP, and no ABL Control: PD ≤ 4 mm, no BOP, and no ABL	*TNF*	Bisulfite-Specific PCR and Pyrosequencing	***TNF*:** Hypermethylated in CP; Increased methylation in CP vs. controls; the overall difference of percentage methylation was marginally significant (*p* = 0.05). No significant difference in methylation pattern in samples biopsied during the induction or resolution phase of experimentally induced gingivitis Site-specific CpG sites (163 and 161) bp: Significantly higher in CP vs. controls at CpG sites -163 (*p* = 0.02) and -161 bp (*p* = 0.04)	CP: A significant inverse association between promoter methylation level at 163 bp and its mRNA expression is present (*p* = 0.018). No such significant association between mRNA expression of *TNF* and promoter methylation at -161 bp was detected Experimental gingivitis: transcription level of *TNF* from biopsies did not exhibit statistical difference among baseline, induction, and resolution phases (*p* = 0.51)	NR	NR	1. CP: PD: 5.7 ± 1.1 mm CAL: 4.1 ± 1.0 mm ABL: Yes 2. Experimental gingivitis: PD: 2.4 ± 0.3 mm CAL: 1.1 ± 0.7 mm ABL: No 2. Controls: PD: 1.9 ± 0.9 mm CAL: 0.9 ± 0.6 mm ABL: No

ABL, alveolar bone loss (*radiographically*); AgP, Aggressive Periodontitis; BOP, bleeding on probing; CAL, clinical attachment level; CCL25, Chemokine (C–C motif) ligand 25; COX2, cyclooxygenase-2; CP, Chronic Periodontitis; CpG, cytosine-guanine; CT, connective tissue; CXCL8, Interleukin 8; ET, epithelia tissue; FADD, Fas associated via death domain; FMBS, full mouth bleeding score; GCF, Gingival crevicular fluid; GT, Gingival tissues; IFNG, interferon gamma; IL10, interleukin-10; IL17C, Interleukin 17C; IL6, interleukin 6; IL6R, interleukin-6 receptor; IRAK1BP1, interleukin-1 receptor-associated kinase 1 binding protein 1; MAP3K7, mitogen-activated protein kinase 7; MMP9, Matrix Metallopeptidase 9; MSP1, merozoite surface protein 1; MSP2, merozoite surface protein 2; MYD88, myeloid differentiation primary response 88; NR, not reported; PB, Peripheral blood; PD, probing depth; PPARA, peroxisome proliferator-activated receptor alpha; PTGS2, Prostaglandin-endoperoxide synthase 2; RIPK2, receptor interacting serine/threonine kinase; SOCS1, suppressors of cytokine signaling 1; SOCS3, suppressors of cytokine signaling 3; TIMP1, Metallopeptidase Inhibitor 1; TLR2, Toll-like receptors 2; TLRs, Toll-like receptors; TNF, tumor necrosis factor.

**Table 3:** Summary of the candidate genes that have been assessed for DNA methylation in periodontitis.

Candidate Genes	DNA Methylation Status in Periodontitis Tissues	Reference
***IFNG***	Non SSD hypermethylation in controls vs. CP; periodontal therapy did not influence gene expression methylation	[22]
	No SSD between CP vs. controls as well as in the experimental gingivitis vs. controls.	[35]
	Hypomethylation in CP vs. controls and experimental gingivitis vs. controls (both SSD).	[19]
***IL6***	Hypomethylation in CP vs. controls; approaching statistical significance.	[31]
	No SSD between moderate vs. CP. Partial methylation was found in both control and CP.	[33]
***IL6R***	Low methylation percentage regardless of the periodontal diagnosis of AgP vs. healthy (Not SSD)	[7]
	Increased methylation in moderate LAP compared to severe LAP (*p* < 0.05) and also when compared to controls.	[30]
***PTGS2***	Hypermethylation in CP vs. controls. An SSD was found among groups in both.	[21,22]
***SOCS1***	No SSD CP vs. controls. There was either no methylation or low methylation status among all groups.	[15]
	SSD was found between groups and a higher percentage of demethylation in controls vs. AgP.	[14]
***TNF***	More methylated in controls vs. CP.	[22,41]
	Methylation status remained stable after two weeks and increased for diseased sites after eight weeks of therapy, approaching baseline for controls; not SSD.	[22]
	No SSD in methylation patterns in experimentally induced gingivitis in vs. resolution phases. Increased methylation in CP vs. controls; significantly (*p* = 0.04). Lower level of methylation in CP vs. control	[36]

AgP, Aggressive Periodontitis; CP, Chronic Periodontitis; IFNG, interferon gamma; IL6, interleukin 6; IL6R, interleukin-6 receptor; LAP, Localized aggressive periodontitis; PTGS2, Prostaglandin-endoperoxide synthase 2; SOCS1, suppressors of cytokine signaling 1; SSD, Statistically significant difference; TNF, tumor necrosis factor.

**Table 4:** Specific gene methylation after periodontal therapy.

Study/Year/Reference	N Cases per Study Group	Type of Periodontal Therapy (Surgical/Non-Surgical)	Type of Tissues	Tissue Harvesting Technique	Methylation Sites	Methods	Methylation Status of Genes at Baseline before Therapy	Methylation Status of Genes after Therapy	Methylation Sites Association between Non-Surgical Therapy and Methylation	Comments
Zhang et al., 2013 [36]	1.CP: 17 2.Experimental gingivitis: 11 3. Controls: 18	1. CP: SRP as initial therapy 4–6 weeks before gingival biopsies’ removal. 2. Experimental gingivitis: Initial dental prophylaxis and supragingival scaling; then experimental gingivitis induced following protocol.	GT 3 biopsies collected at 1 week following therapy (baseline), at day 21 following the induction period, and at 4 weeks after resuming DH practice (resolution phase).	NR	*TNF* CpG sites-163 and -161 bp	Bisulfite-Specific PCR and Pyrosequencing	Levels assessed but NR	Levels assessed during resolution phase 4 weeks after resuming dental hygiene practice but NR	*TNF*: No significant difference in methylation during the induction compared to resolution phases of experimentally induced gingivitis.	The transcription level of *TNF* from biopsies in the experimentally induced gingivitis study did not exhibit statistical difference among baseline, induction, and resolution phases (*p* = 0.51).
Andia et al., 2015 [15]	1.CP: 10 2. Controls: 10	1&2. CP and controls: Full-mouth prophylaxis and removal of supragingival calculus/biofilm retentive factors and biofilm, and condemned teeth extractions. 2. CP only: Subgingival instrumentation. Surgical procedures were carried Out at three months after basic therapy, in the areas with remaining periodontal pockets. Recall visits, including supragingival prophylaxis and the reinforcement of oral hygiene, occurred monthly up to three months.	GT 1. CP: gingival biopsies collected 3 months after controlling inflammation	Laser capture microdissection	*SOCS3*, *SOCS1*	Bisulfite restriction analysis combined with Methylation Specific- High Resolution Melting Analysis	NR	*SOC1* and *SOC3* methylation status: No differences found between controlled CP three months following periodontal therapy and controls	*SOCS1*, *SOCS3*: Three months following control of inflammation in GT, ET methylation profile was similar CT from patients that were previously affected or not by chronic inflammation.	NR
Asa’ad et al., 2017 [22]	1. CP: 10 2. Controls: 10	1. CP: Full-mouth SRP with ultrasonic and manual instruments. Chlorhexidine mouthwash (0.2%) prescribed for daily use (twice daily for 20 days)	GT 1. CP: Gingival biopsies collected at baseline and 2 and 8 weeks following periodontal therapy; normal and periodontal sites biopsied	NR	*COX2*, *IFNG*, *TNF*	Pyrosequencing	*TNF* methylation: Higher in controls vs. CP (normal and periodontal sites) at baseline 1. *TNF* methylation CP: 31.4% (7.8) in periodontitis sites and 34.1% (5.2) in normal sites 2. *TNF* methylation controls: 36.6% (9.2) at baseline	*TNF* methylation in CP: Almost stable throughout evaluation period in normal sites vs. almost stable up to two weeks and started to rise—reaching 33.7% (7.4) at 8 weeks in periodontitis sites; periodontitis sites at 8 weeks similar to normal sites at baseline but not statistically significant.	*TNF*, *IFNG*: Periodontal therapy did not influence gene expression methylation levels in CP and controls over time.	NR
							*IFNG* methylation: Highest in controls and almost comparable to controls in CP periodontitis sites, but slightly lower methylation in CP normal sites 1. *IFNG* methylation CP: 88.3% (2.1) in periodontitis sites and 85.8% (6.5) in normal sites 2. *IFNG* methylation controls: 88.6% (1.4)	*IFNG* methylation in CP: Started to decline in periodontitis sites and percentage at 8 weeks (86.2% (3.8)) almost equivalent to normal sites at baseline; methylation levels in normal sites started to increase approaching a level at 8 weeks (88.8% (2.8) comparable to controls. None of these findings was statistically significant (*p*-value > 0.05).		
							*COX2* mean methylation: Nearly twice as high in CP periodontitis sites vs. controls with a statistically significant difference in CP periodontitis sites vs. controls (*p* = 0.03) at baseline 1. *COX2* methylation CP: 13.2% (7.3) in periodontitis sites vs. 8.8% (5.7) in normal sites 2. *COX2* methylation controls: 6.7% (7.6)	*COX2* methylation in CP: Methylation percentage decreased dramatically in periodontitis sites following periodontal therapy, at two weeks, almost to half (5.8% (3.2), *p* < 0.001 versus baseline), with a very slight rise from 2 to 8 weeks (7.2% (4.3), *p* = 0.004 versus baseline) and comparable to the methylation level reported in controls; mean percentage of methylation steadily declined in CP normal sites, reaching 5% (1.9 at 8 weeks, *p* = 0.03 versus baseline).	*COX2*: Significantly reduced *COX2* methylation levels comparable to healthy individuals at both 2- and 8-weeks post-treatment (*p* < 0.05).	

ABL, alveolar bone loss (*radiographically*); AgP, Aggressive Periodontitis; BOP, bleeding on probing; CAL, clinical attachment level; COX2, cyclooxygenase-2; CP, Chronic Periodontitis; CpG, cytosine-guanine; CT, connective tissue; ET, epithelial tissue; GCF, Gingival crevicular fluid; GT, Gingival tissues; IFNG, interferon gamma; NR, not reported; PB, Peripheral blood; SOCS1, suppressors of cytokine signaling 1; SOCS3, suppressors of cytokine signaling 3;TNF, tumor necrosis factor.

## Supplementary Materials

The following are available online at https://www.mdpi.com/1422-0067/21/17/6217/s1.
