# Supplementary material for: The Role of DNA Methylation and Histone Modification in Periodontal Disease: A Systematic Review
Source: Int J Mol Sci. 2020 Aug 27;21(17):6217. doi: 10.3390/ijms21176217 (PMC7503325; doi:10.3390/ijms21176217)
Supplement: Supplementary file 1 [file ijms-21-06217-s001.zip › Table S1.docx]

**Table S1.** Database searches.

The following search terms, created by a medical-dental librarian, were used for database searches.

| PUBMED | ((Epigenomics[mh] OR DNA methylation[mh] OR S-Adenosylmethionine[mh] OR CpG Islands[mh] OR ((histone*[tiab] OR dna OR long interspersed) AND (acetylat*[tiab] OR demethylat*[tiab] OR methylat*[tiab] OR phosphorylat*[tiab] OR ubiquitinat*[tiab] OR modif*[tiab]) OR s adenosylmethionine OR cpg OR epigenetic*[tiab])) AND ("periodontal diseases"[MeSH Terms] OR ("periodontal"[All Fields] AND "diseases"[All Fields]) OR "periodontal diseases"[All Fields] OR ("periodontal"[All Fields] AND "disease"[All Fields]) OR "periodontal disease"[All Fields] OR "periodontitis"[MeSH Terms] OR "periodontitis"[All Fields])) |
| --- | --- |
| EMBASE (OVID) | (Epigenomics/ or DNA methylation/ or S-Adenosylmethionine/ or CpG Islands/ or (((histone* or dna or long interspersed) adj3 (acetylat* or demethylat* or methylat* or phosphorylat* or ubiquitinat* or modif*)) or s adenosylmethionine or cpg or epigenetic*).ab,ti.) and (exp periodontal disease/ or periodontal disease.mp. or periodontal diseases.mp. or periodontitis.mp. or exp periodontitis/) |
| MEDLINE (OVID) | (exp Epigenomics/ OR exp DNA Methylation/ OR exp S-Adenosylmethionine/ OR exp CpG Islands/ OR (((histone* OR dna OR long interspersed) ADJ3 (acetylat* OR demethylat* OR methylat* OR phosphorylat* OR ubiquitinat* OR modif*)) OR s adenosylmethionine OR cpg OR epigenetic*).ab,ti.) AND (exp Periodontal Diseases/ or periodontal diseases.mp. or periodontal disease.mp. or periodontitis.mp. or exp PERIODONTITIS/) |
| CINAHL (VIA EBSCO) | ((MH Epigenomics+ OR MH "DNA methylation+" OR MH "S-Adenosylmethionine+" OR (((histone* OR dna OR long interspersed) N3 (acetylat* OR demethylat* OR methylat* OR phosphorylat* OR ubiquitinat* OR modif*)) OR s-adenosylmethionine OR cpg OR epigenetic*)) AND (periodontal disease OR MH periodontal diseases OR periodontitis OR MH periodontitis)) |
| WEB OF SCIENCE | TS=((((histone* OR dna OR "long interspersed") NEAR/3 (acetylat* OR demethylat* OR methylat* OR phosphorylat* OR ubiquitinat* OR modif*)) OR "s adenosylmethionine" OR cpg OR epigenetic* OR epigenomic*)) AND TS=((periodontal disease) or (periodontal diseases) or (periodontitis)) |
| SCOPUS | TITLE-ABS-KEY(((((histone* OR dna OR "long interspersed") W/3 (acetylat* OR demethylat* OR methylat* OR phosphorylat* OR ubiquitinat* OR modif*)) OR "s adenosylmethionine" OR cpg OR epigenetic* OR epigenomic*)) AND ((periodontal disease) OR (periodontaldiseases) OR (periodontitis))) |
| SCIELO | TS=((((histone* OR dna OR "long interspersed") NEAR/3 (acetylat* OR demethylat* OR methylat* OR phosphorylat* OR ubiquitinat* OR modif*)) OR "s adenosylmethionine" OR cpg OR epigenetic* OR epigenomic*)) AND TS=((periodontal disease) or (periodontal diseases) or (periodontitis)) |
