# Supplementary material for: The Role of DNA Methylation and Histone Modification in Periodontal Disease: A Systematic Review
Source: Int J Mol Sci. 2020 Aug 27;21(17):6217. doi: 10.3390/ijms21176217 (PMC7503325; doi:10.3390/ijms21176217)
Supplement: Supplementary file 1 [file ijms-21-06217-s001.zip › Table S2.docx]

Table S2. Extracted information on study characteristics.

| **Extracted Information on Study Characteristics** |
| --- |
| Publication Details (Author (s), Year of Publication) |
| Study Methods/Study Design |
| Study Period |
| Location |
| Sample Size |
| Age and Gender of Participants |
| Population (diagnostic criteria), |
| Duration of Follow up (for longitudinal studies), |
| Confounders |
| Tissue Sample |
| Assays and Techniques Performed |
| Outcome |
| Controls |
| Major Findings |
| Significant Difference Outcomes |
| Statistical Method Used in the Analysis. |

Data were extracted by two review authors (RB, MM) using a data form that was specifically designed for the present systematic review and modified for second review process as required for presentation of additional general characteristics and secondary outcomes. All data and data forms were reviewed twice by both authors. A third party (IK) moderated any disagreement when needed. All study authors were contacted for clarification of methodology/results and missing information.
