# Supplementary material for: The Role of DNA Methylation and Histone Modification in Periodontal Disease: A Systematic Review
Source: Int J Mol Sci. 2020 Aug 27;21(17):6217. doi: 10.3390/ijms21176217 (PMC7503325; doi:10.3390/ijms21176217)
Supplement: Supplementary file 1 [file ijms-21-06217-s001.zip › Table S3.docx]

**Table 3.** Studies Excluded After Full-text Review.

| **Reference** | **Excluded study** | **Reason for exclusion** |
| --- | --- | --- |
| [1] | (Schulz 2014) | Title only; neither full text articles nor contact info for corresponding authors found |
| [2] | (Winfield, Esbitt et al. 2016) | Meeting abstract only; neither full text articles nor contact info for corresponding authors found |
| [3] | (Richter, Kruppa et al. 2019) | No comparison of periodontitis to control; focused on periodontally healthy patients only. |

**References:**

1. Schulz, S.S., H. G.; Immel, U.D.; Just, L.; Glaeser, C.; Reichert, S. Epigenetic characteristics in inflammatory candidate genes in aggressive periodontitis: The role of interleukin 17C. Medizinische Genetik 2014, 26, 177.

2. Winfield, J.; Esbitt, A.; Seutter, S.F.; Desai, B.; Abdo, M.; Vasconez, M.; Laidlaw, W.; Green, K.; Shamseddin, S.M.; Borghaei, R.C. Effect of Inflammatory Cytokines on DNA Methylation and Demethylation. The FASEB Journal 2016, 30, 1053.1053-1053.1053, doi:10.1096/fasebj.30.1_supplement.1053.3.

3. Richter, G.M.; Kruppa, J.; Munz, M.; Wiehe, R.; Hasler, R.; Franke, A.; Martins, O.; Jockel-Schneider, Y.; Bruckmann, C.; Dommisch, H., et al. A combined epigenome- and transcriptome-wide association study of the oral masticatory mucosa assigns CYP1B1 a central role for epithelial health in smokers. Clinical epigenetics 2019, 11, 105, doi:10.1186/s13148-019-0697-y.
