# Supplementary material for: The Role of DNA Methylation and Histone Modification in Periodontal Disease: A Systematic Review
Source: Int J Mol Sci. 2020 Aug 27;21(17):6217. doi: 10.3390/ijms21176217 (PMC7503325; doi:10.3390/ijms21176217)
Supplement: Supplementary file 1 [file ijms-21-06217-s001.zip › Table S4.docx]

**Table 4.** Risk of Quality Assessment. **.**

| Item Number |  | **Andia et al. 2010 [18]** | **Andia et al. 2015 [15]** | **Asa'ad et al. 2017 [22]** | **Baptistat et al. 2014 [14]** | **de Faria et al. 2013 [34]** | **de Souza et al. 2014 [32]** | **Kobayashi et al. 2016 [31]** | **Lavu et al. 2019 [41]** | **Li et al. 2018 [29]** | **Schulz et al. 2016 [7]** | **Shaddox et al. 2017 [30]** | **Stefani et al. 2013 [33]** | **Viana et al. 2011 [35]** | **Zhang, Barros et al. 2010 [21]** | **Zhang, Crivello et al. 2010 [19]** | **Zhang et al. 2013 [36]** |
| --- | --- | --- | --- | --- | --- | --- | --- | --- | --- | --- | --- | --- | --- | --- | --- | --- | --- |
| 1 | Clearly stated research question/objective? | Yes | Yes | Yes | Yes | Yes | Yes | Yes | Yes | Yes | Yes | Yes | Yes | Yes | Yes | Yes | Yes |
| 2 | Clearly specified and defined study population? | Yes | Yes | Yes | Yes | No | No | Yes | Yes | No | Yes | Yes | No | No | No | No | No |
| 3 | Participation rate of eligible persons at least 50%? | Yes | Yes | Yes | Yes | Yes | Yes | Yes | Yes | Yes | Yes | Yes | Yes | Yes | Yes | Yes | Yes |
| 4 | Subjects selected from the same/similar populations? Were inclusion/exclusion criteria for prespecified and applied uniformly to all participants | Yes | Yes | Yes | Yes | Yes | Yes | Yes | Yes | Yes | Yes | Yes | No | Yes | Yes | Yes | Yes |
| 5 | Sample size justification, power description/variance and effect estimates were provided? | No | No | No | No | No | No | No | Yes | No | No | No | No | No | No | No | No |
| 6 | Exposure(s) of interest measured prior to the outcome(s)? | No | No | Yes | No | No | No | No | Yes | No | No | No | Yes | No | No | Yes | Yes |
| 7 | Sufficient timeframe to see an association between exposure and outcome if it existed? | Yes | Yes | Yes | Yes | Yes | Yes | Yes | Yes | Yes | Yes | Yes | Yes | Yes | Yes | Yes | Yes |
| 8 | For varying amounts of exposure, were different levels of the exposure as related to the outcome examined? | No | No | No | No | No | No | No | No | Yes | No | Yes | No | No | No | No | No |
| 9 | Exposure measures were clearly defined, valid, reliable and implemented consistently across all study participants? | Yes | Yes | Yes | Yes | Yes | Yes | Yes | Yes | Yes | Yes | Yes | Yes | Yes | Yes | Yes | Yes |
| 10 | The exposure(s) assessed more than once over time? | No | No | Yes | No | No | No | No | No | No | No | No | No | No | No | No | Yes |
| 11 | Outcome measures were clearly defined, valid, reliable and implemented consistently across all study participants? | Yes | Yes | Yes | Yes | Yes | Yes | Yes | Yes | Yes | Yes | Yes | Yes | Yes | Yes | Yes | Yes |
| 12 | Outcome assessors blinded to the exposure status of participants? | No | No | No | No | No | No | No | No | No | No | No | No | No | No | No | No |
| 13 | Loss to follow-up after baseline 20% or less? | NA | NA | Yes | NA | NA | NA | NA | NA | NA | NA | NA | NA | NA | NA | NA | NR |
| 14 | Were key potential confounding variables measured and adjusted statistical for their impact on the relationship between exposure(s) and outcome(s)? | No | No | Yes | No | No | No | No | No | No | Yes | Yes | No | No | No | No | No |
|  | Overall (Good, fair, poor) | Fair | Fair | Good | Fair | Poor | Poor | Fair | Good | Fair | Fair | Good | Poor | Poor | Poor | Fair | Fair |

**Table Legend**

NA, not applicable; NR, not reported;

Overall score (yes) >8: Good; (yes)7 to 8: Fair; <7: Poor
