# Supplementary material for: The Role of DNA Methylation and Histone Modification in Periodontal Disease: A Systematic Review
Source: Int J Mol Sci. 2020 Aug 27;21(17):6217. doi: 10.3390/ijms21176217 (PMC7503325; doi:10.3390/ijms21176217)
Supplement: Supplementary file 1 [file ijms-21-06217-s001.zip › Figure S1.doc]

**Figure S1.** PRISMA Flow Diagram.

**Screening**

**Included**

**Eligibility**

**Identification**

Records identified through database searching
(n = 1221)

Additional records identified through other sources
(n = 1)

Records after duplicates removed
(n = 523)

Records screened
(n = 523)

Records excluded
(n = 502)

Full-text articles assessed for eligibility
(n = 21)

Full-text articles excluded
(n = 5)

Studies included in qualitative synthesis
(n = 16)

Studies included in quantitative synthesis (meta-analysis)
(n = 0)
